# Perioperative Anxiety: Current Status and Future Perspectives

**DOI:** 10.3390/jcm14051422

**Published:** 2025-02-20

**Authors:** Corina Manuela Bello, Patryk Eisler, Thomas Heidegger

**Affiliations:** 1 Clinic of Anaesthesiology, Intensive Care Medicine and Pain Therapy, Hirslanden Klinik St. Anna Lucerne, 6006 Luzern, Switzerland; 2 Klinik für Anästhesiologie, Intensiv- und Rettungsmedizin, HOCH Health Ostschweiz, Spital Grabs, 9472 Grabs, Switzerland; patryk.eisler@h-och.ch (P.E.); thomas.heidegger@h-och.ch (T.H.); 3 Department of Anaesthesiology and Pain Medicine, Inselspital, Bern University Hospital, University of Bern, 3012 Bern, Switzerland

**Keywords:** preoperative anxiety, perioperative care, anesthesia, quality of care

## Abstract

Perioperative anxiety is a multifaceted phenomenon that significantly impacts patients undergoing surgical procedures. Despite advancements in surgical techniques and anesthetic management, the psychological burden associated with anesthesia and surgery remains a significant challenge for healthcare providers. Up to 30% of patients suffer from anesthesia-related preoperative anxiety, irrespective of whether the procedure is elective or emergent. Notably, anxiety can adversely affect patient outcomes, including pain management, patient safety, overall quality of care, and patient satisfaction. Addressing perioperative anxiety requires a comprehensive understanding of its causes, assessment tools, and management strategies to ensure optimal perioperative care. This review examines the historical context, incidence, causes, pathophysiology, assessment tools, and current evidence regarding management strategies for anesthesia-related anxiety, and provides an outlook on future directions for research and everyday practice.

## 1. Introduction

### 1.1. Historical Background

Currently, up to 30% of patients experience preoperative anxiety related to anesthesia, regardless of whether the procedure is elective or emergent emergent [1]. Throughout history, surgical procedures have been accompanied by varying degrees of patient anxiety, influenced by evolving medical practices and societal perceptions of anesthesia. Early anxiety management involved physical restraint and substances like alcohol, which had limited success [2,3]. The introduction of ether and chloroform in the mid-19th century improved physical comfort but did not address the psychological dimensions of anxiety. By the mid-20th century, benzodiazepines and sedatives became common for managing anxiety. These agents were frequently employed as premedication to reduce anxiety, induce amnesia, and mitigate the physiological effects of heightened sympathetic activity, thereby enhancing the safety of anesthesia [4,5,6]. In the late 20th century, non-pharmacological approaches, such as relaxation techniques [7], gained attention, and patient education tools, like video-based materials [8], helped reduce preoperative fear. The importance of a multidisciplinary, patient-centered approach has grown [9,10], though significant gaps remain in understanding and managing preoperative anxiety.

### 1.2. Incidence and Causes of Perioperative Anxiety

Approximately one-third of surgical patients experience anesthesia-related anxiety [1], with higher prevalence observed in specific subgroups such as women and the elderly [11,12,13,14,15,16,17,18]. Moreover, certain specialties of surgery show a higher incidence of anxiety (e.g., gynecologic surgery [15,19]). Anxiety levels often fluctuate throughout the perioperative timeline, with fears shifting from anesthesia-related concerns preoperatively to pain and complications postoperatively. Understanding these dynamics is essential for tailoring interventions to individual patients.

Anesthesia-specific anxiety involves fears of loss of control over oneself, waking up during surgery (i.e., intraoperative awareness), the inability to wake post-intervention, fear related to an anesthetist’s error, fear of pain, or fear of postoperative nausea and vomiting. Among these causes of anxiety, the fear of not waking up is the most reported [12,18,20,21,22,23].

Several factors contribute to the incidence and severity of perioperative anxiety, including demographic, psychological, medical, social, and informational influences. Demographic factors such as age and gender play a role, with children, elderly patients, and women being more susceptible [20]. Psychological predispositions, including pre-existing anxiety disorders also contribute significantly [24]. A profound understanding of perioperative anxiety is essential for tailoring interventions to individual patients.

### 1.3. Pathophysiology

The pathophysiology of perioperative anxiety involves both psychological and physiological components. Elevated sympathetic activity increases cortisol levels, heart rate, and blood pressure, which complicate anesthetic induction and maintenance [25,26]. Moreover, this heightened physical stress response has been linked to a cascade of adverse events following anesthesia. These include postoperative nausea and vomiting, pain, insomnia, neurocognitive dysfunction, delayed recovery, prolonged hospital stays, and even long-term mortality [27]. Addressing these physiological manifestations is crucial to optimizing patient outcomes.

## 2. Assessment Tools

### Questionnaires, Scales, and Scores

Standardized tools are commonly used to assess anxiety. They include the Amsterdam Preoperative Anxiety and Information Scale (APAIS), the State-Trait Anxiety Inventory (STAI), and the Hospital Anxiety and Depression Scale (HADS). APAIS is a validated instrument to measure patient anxiety and information needs [16]. STAI allows for differentiation between situational and baseline anxiety [28]. HADS identifies anxiety and depressive symptoms in hospitalized patients [29]. While these tools provide valuable insights, their limitations in perioperative settings—such as the lack of consensus on a clinically significant threshold for anesthesia-related anxiety—highlight the need for more specific instruments tailored to surgical contexts. Moreover, they are very time-consuming. Visual analog scales and numeric rating scales, though simple and quick, often fail to capture the nuanced causes and implications of perioperative anxiety [30].

## 3. Management Strategies

Sixty-five percent of patients suffering from perioperative anxiety demand support from the anesthetist in coping with distressing feelings [30]. The management of the multifaceted phenomenon of perioperative anxiety asks for a holistic and preemptive approach.

### 3.1. Pharmacological Interventions

Benzodiazepines, such as midazolam, remain the cornerstone of pharmacological management, providing rapid anxiolysis and, therefore, enabling practitioners to relieve anxiety even when it is only identified upon entering the operating or induction room. However, their sedative effects necessitate careful dosing and monitoring [5,31]. Moreover, there are still controversies concerning an increased risk of postoperative delirium in patients who received midazolam and other benzodiazepines. Despite some evidence suggesting no disadvantageous effect of midazolam on postoperative cognitive function in patients > 70 years of age [32], current guidelines advise against the use of benzodiazepines in the whole perioperative period in elderly patients in order to prevent delirium and cognitive dysfunction [33,34,35]. Hence, a recommendation to use these drugs to mitigate anxiety cannot be made based on today’s evidence.

Gabapentinoids provide both anxiolysis and analgesia [36]. These drugs have been shown to reduce perioperative opioid requirements; however, this effect varies among different agents. As an example, gabapentin could not alleviate anxiety compared with placebo in patients undergoing abdominal hysterectomy, whereas administering alprazolam pre-operatively appeared to be beneficial. Both drugs showed no effect on postoperative morphine consumption [37]. The findings on whether gabapentinoids are useful to alleviate anxiety, and even mitigate postoperative pain, remain controversial, and adverse side effects, such as sedation upon administration of these drugs, should be carefully considered in daily practice [37].

Beta-blockers (e.g., propranolol) have shown efficacy in mitigating the physiological manifestations of anxiety, such as tachycardia and hypertension [38,39]. They come without the risk of sedation compared with benzodiazepines and gabapentinoids, and show a comparable effect on anxiety levels to the above-mentioned groups of drugs [40]. Still, hypotension and bradycardia are extremely dangerous, notable side effects of these drugs, extending way beyond the intraoperative period. Administered to patients with cardiac risk factors or in higher doses, they may cause major cardiac events up to 30 days after surgery [41] and should be used with caution, if at all. Hence, the effectiveness of pharmacological interventions to reduce anxiety while safeguarding patients’ safety remains limited [42].

In recent years, melatonin and dexmedetomidine have emerged as effective alternatives to benzodiazepines for managing preoperative anxiety. Melatonin is a physiologic sleep–wake cycle regulator. Its sedative and anxiolytic properties make it an effective alternative to benzodiazepines for managing preoperative anxiety. Melatonin significantly reduces preoperative anxiety while having less impact on psychomotor and cognitive function than benzodiazepines [31,43]. Melatonin’s effects on anxiety levels have even been shown in women who have a high incidence of perioperative anxiety [44]. The findings in children are still inconclusive [45]. Dexmedetomidine on the other hand, selectively acts as an agonist at the α2-adrenergic receptors. It has sedative, analgesic, and anxiolytic effects. Besides the intravenous application, it can be given nasally, making it a potent anxiety-reducing drug for children in whom no IV access has been established [46]. Furthermore, it acts faster and more predictably than oral midazolam [47].

### 3.2. Non-Pharmacological Management

Non-pharmacological strategies offer valuable alternatives to manage perioperative anxiety without the risks of medications. Cognitive behavioral therapy (CBT) helps patients reframe anxiety-inducing thoughts, fostering a sense of control and reducing anxiety [48,49]. Techniques like aromatherapy, particularly with lavender, have been shown to modestly decrease anxiety levels in preoperative settings [50,51]. Additionally, methods such as hypnosis and guided imagery effectively calm patients [52,53], while massage promotes relaxation and reduces anxiety [54]. Acupuncture has also been explored, with some studies suggesting it can provide relief from preoperative anxiety, making this approach valuable for enhancing patient well-being before surgery [55].

However, these methods often require additional resources and expertise, underscoring the need for broader implementation strategies and a multidisciplinary approach.

Anesthetists and surgeons may still be able to address anxiety by other, more readily available, options. Listening to calming music or music of the patient’s choice before surgery has demonstrated significant reductions in anxiety levels in patients undergoing spinal anesthesia [56]. Immersive virtual reality (VR) experiences provide distraction and relaxation, effectively reducing preoperative anxiety [57,58]. A recently published comprehensive review provides a profound discussion of the evidence supporting non-pharmacological management strategies [59].

### 3.3. Information-Based Interventions

An effective strategy to alleviate preoperative anxiety is thorough consultation before surgery, especially by anesthesiologists [10]. Notably, the timing of these consultations plays a pivotal role in their effectiveness, with an ideal timepoint at two weeks prior to surgery [60]. Furthermore, the content and means of transmission of information are of importance. Anesthetist staff shortages, time constraints, and an increasing lack of contact with patients early enough before anesthesia/surgery limit this cornerstone of anxiety management. It is here that integrating modern technologies such as video tutorials, virtual reality simulations, and digital anesthesia service platforms may improve communication and provide personalized education for patients. A video-based pre-anesthesia information transfer has been shown to be effective in reducing anxiety both as a stand-alone intervention or in combination with an “empathic” pre-anesthesia interview-style by “trained” anesthetists, compared with the standard of care [10]. Interestingly, providing the patients with video-based information also significantly reduced the interview time while decreasing the need for premedication and the level of anxiety on the day of surgery. This is an important finding, especially in times when healthcare providers suffer from time pressure. Multimedia tools are particularly beneficial in tailoring information to each patient’s unique circumstances, including factors like age, educational background, gender, and the nature of the planned surgery. By addressing specific concerns and uncertainties, these approaches not only help minimize preoperative anxiety but may also contribute to a reduction in postoperative complications, which require further investigation.

## 4. Special Settings

### 4.1. Pediatric Anesthesia

Children are particularly vulnerable to perioperative anxiety. Identifying perioperative anxiety in children has been recognized as a major challenge for anesthesia providers [61]. Several scales are available for research purposes but have limited clinical application [62,63]. Simplified scales such as the “Pediatric Anesthesia Behavior” (PAB) tool or the “Happy, Relaxed, Anxious, Distress with yes/no cooperation” (HRAD±) have been developed instead [64,65]. Although easy to use, so far, they lack broad validation.

Pharmacological options remain limited, as newer medications have not proven superior to benzodiazepines [45,66]. Alpha-2 agonists (clonidine, dexmedetomidine) are effective [67]. However, long onset times and the risk of bradycardia make them suboptimal in the perioperative setting.

Non-pharmacological strategies, such as distraction techniques or parental presence, help mitigate anxiety in this population [68]. Interestingly, integrating multiple non-pharmacological methods acts synergistically [68].

Caregivers (parents or guardians) are also susceptible to anxiety in the preoperative period, which can have a profound impact on the child’s stress levels. Parental anxiety can amplify the child’s baseline stress level, creating a cyclical effect where both the child and caregiver experience increased anxiety. Several studies highlight the importance of social factors in preoperative preparation, including effective communication strategies to manage anxiety among both children and their caregivers [69,70].

A recently published clinical focus review on the topic of pediatric perioperative anxiety provides deeper insight into this specific patient group [71].

### 4.2. Day-Case Surgery

The fast-paced environment of day-case surgery presents unique challenges for anxiety management [72]. While intended to be a calm experience for patients with the benefit of quickly returning to the comfort of their homes, the stress-response to day-case surgery is highly underrated [26]. Preoperative pharmacological interventions and concise, focused patient education can significantly improve patient experiences in these settings (Table 1).

## 5. Patient Safety, Quality of Care, and Anxiety

Effective management of anxiety plays a crucial role in ensuring patient safety. Elevated anxiety levels can lead to increased sympathetic activity, resulting in tachycardia, hypertension, and other physiological changes that may complicate anesthetic induction and surgical outcomes. By addressing anxiety, anesthesiologists can minimize these risks, contributing to safer surgical experiences [27]. Non-pharmacological approaches, such as preoperative counseling, relaxation techniques, and patient education, complement pharmacological interventions and have been shown to enhance both safety and satisfaction [59]. Patient satisfaction is intrinsically linked to the perceived quality of anesthesia [15]. Quality of anesthesia includes the reduction in pain, the absence of adverse effects, and the management of anxiety before and after surgery. Quality in anesthesia is defined by six domains: effectiveness, equity, timeliness, efficiency, safety, and patient-centeredness. Patient-centeredness highlights the need to respect and respond to individual patient preferences, needs, and values [73]. High levels of anxiety can negatively impact patient satisfaction both directly [15] and by heightening the perception of pain, increasing postoperative complications, and prolonging recovery time [74,75]. The integration of patient-centered strategies, including tailored communication and psychological support, underscores the shift towards holistic anesthesia care. By prioritizing both the psychological and physical well-being of patients, contemporary anesthetic practices aim to optimize patient satisfaction, ensure safety, and deliver high-quality care that addresses the multifaceted nature of perioperative anxiety.

## 6. Future Directions

**Figure 1:**
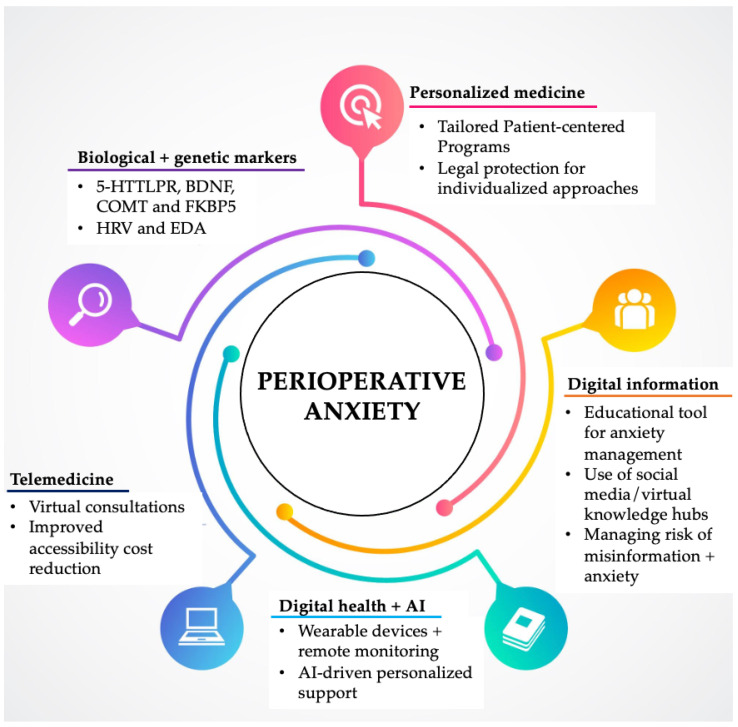
Future directions for managing perioperative anxiety (design by Freepik).

The future of perioperative anxiety management is entering an exciting era, where AI-driven screening and support merge with VR-based patient education, personalized pharmacological regimens, integrated mental health solutions, and dynamic biometric monitoring to revolutionize the entire surgical journey—from the initial consultation to recovery (Figure 1). Advancement in anxiety assessment methods and measurements can further improve the detection of patients in need of intervention. Furthermore, differentiating between perioperative stress and anxiety needs to be addressed.

### 6.1. Biological and Genetic Markers

The pre-surgery period is often marked by the progression of pathology, while the anticipation of anesthesia and surgery heightens stress on the body. Research into the interaction of these factors offers valuable insights into the origins and mechanisms of perioperative anxiety. Bespoke medication strategies can reduce overall anxiety levels and minimize side effects. Biochemical and physiological studies can bring better understanding of processes occurring in the human body when experiencing perioperative anxiety. Research in neurophysiology and brain plasticity can help us understand the interplay between stress hormones and brain recovery mechanisms [76]. In addition, emerging evidence suggests that genetic and biological markers, such as variations in the serotonin-transporter-linked promoter region (5-HTTLPR) gene [77] and increased inflammatory markers [78], may predict susceptibility to anxiety. The brain-derived neurotrophic factor (BDNF), catecholamine-O-methyltransferase (COMT), and FK506-binding protein 5 (FKBP5)-gene are other possible genetic markers for altered anxiety levels [79,80,81]. Ultimately, researchers have explored a link between lower heart-rate-variability (HRV) and the presence of anxiety [82]. With HRV-monitoring tools at hand via smartphone applications, these might help identify patients experiencing higher-than-normal stress. Another biological marker with potential to indicate stress is electrodermal activity (EDA). Biomarker research in non-clinical settings has shown encouraging results regarding correlation with established stress questionnaires [83]. While promising, these findings currently have limited clinical application and are primarily used in research or in e-health applications. Future studies should focus on integrating these markers into practical, predictive models for perioperative care.

### 6.2. Digital Health and Artificial Intelligence

Wearable e-health devices can lead to tailored pre- and postoperative recommendations and treatment plans, reducing anxiety related to surgical procedures. Wearable medical devices and remote monitoring, together with advancements in machine learning, will bring possibilities of personalized interaction with specially developed artificial intelligence systems, giving access to high-quality information and tailored support and treatment solutions [84].

### 6.3. Digital Information

When it comes to information, social media represents both a valuable resource and a potential obstacle in the pursuit of a solution. On the one hand, it may serve as a readily available information-distributing tool. On the other hand, the unfiltered flow of information without appropriate context can increase anxiety [85].

### 6.4. Telemedicine

The number of studies examining telephone-based anesthesia consultation doubled in the years following the COVID-19 pandemic. The immediate need to reduce patient contact with healthcare providers evolved into the realization that certain patient groups can be successfully assessed and receive information prior to anesthesia without needing to travel to the hospital. High patient satisfaction, a reduced carbon footprint, and reduced costs are some of the benefits connected to telephone anesthesia evaluation [86,87].

### 6.5. Personalized Medicine

Sociocultural contributing factors of perioperative anxiety must be further explored. Perioperative anxiety has been repeatedly shown to affect up to 1.27 times as many women as men [18,88]. Similarly, social and sociodemographic differences contribute to a higher incidence of perioperative anxiety among patient subpopulations [89]. Understanding the underlying mechanisms is crucial to develop appropriate strategies for better outcomes. As globalized societies develop, health care providers need to increase their sensitivity about aspects of cultural subtleties to personalize the patient experience.

Patient-centered programs, developed for certain groups based on gender, age, or special needs, and created by multidisciplinary teams including physicians, psychologists, and other experts can allow leaving the one-fits-all paradigm of preventing and treating perioperative anxiety. This further includes a personalized preoperative information strategy, accepting that the need for preoperative information varies significantly, and legally protecting anesthesiologists who act accordingly.

## 7. Conclusions

Perioperative anxiety is very common. Integrating a multidisciplinary approach, combined with pharmacological, psychological, and technological strategies, is of utmost importance. Anesthesiologists and surgeons should collaborate with other specialists to design comprehensive care plans that address the multifaceted nature of perioperative anxiety. More personalized options, including tailored means to convey information, may not only empower patients but also optimize healthcare resource utilization, making them a key area for future innovation. Further research is thus urgently needed to identify and manage perioperative anxiety more effectively.

## Figures and Tables

**Table 1:** Summary of assessment scales, risk factors, and potential management strategies of perioperative anxiety and their effectiveness.

Category	Name	Advantages (pro) and Considerations (con)
**Assessment tool**	APAIS	**Pro**: Validated for measuring anxiety and information needs. **Con**: Time-consuming, lacks specificity for anesthesia-related anxiety.
	STAI	**Pro**: Differentiates between situational and baseline anxiety. **Con**: Requires considerable time and effort to administer.
	HADS	**Pro**: Identifies anxiety and depression in hospitalized patients. **Con**: Limited use in perioperative contexts, lacks clear thresholds for anxiety.
	VAS	**Pro**: Simple and quick. **Con**: Fails to capture nuanced causes and implications of perioperative anxiety.
**Risk factors**	Gender (Female), Age (Elderly), Surgical Specialty (e.g. Gynecology)	**Pro:** Certain patient populations/surgical procedures show higher rates of perioperative anxiety. **Con**: Anxiety is linked to various factors and may result from multiple causes. Broad categories might miss individual patient needs.
**Therapeutic option**	Benzodiazepines (e.g. Midazolam)	**Pro**: Rapid anxiolysis, effective in acute situations. **Con**: Risk of postoperative delirium (elderly patients).
	Gabapentinoids (e.g. Gabapentin)	**Pro**: Provides both anxiolysis and analgesia. **Con**: Mixed results, some agents ineffective for anxiety relief, possible side effects like sedation.
	Beta-blockers (e.g. Propranolol)	**Pro**: No sedation, effective for physiological symptoms like tachycardia. **Con**: Risk of hypotension, bradycardia, and cardiac events.
	Melatonin	**Pro**: Sedative and anxiolytic properties, less cognitive impact than benzodiazepines. **Con**: Limited evidence in some populations (e.g. children).
	Dexmedetomidine	**Pro**: Anxiolytic, analgesic, and sedative effects; effective in children. **Con**: Intravenous or nasal administration, costly, slower onset.
	Cognitive Behavioral Therapy	**Pro**: Reframes anxiety-inducing thoughts, promotes control and relaxation. **Con**: Requires time, resources, and expertise.
	Music Therapy	**Pro**: Non-invasive, reduces anxiety significantly. **Con**: Requires access to equipment and time for preparation.
	Aromatherapy	**Pro**: Easy to administer, shown to reduce anxiety in some studies. **Con**: Limited evidence base, effectiveness may vary across individuals.
	Virtual Reality (VR)	**Pro**: Provides immersive distraction and relaxation, effective in reducing anxiety. **Con**: Requires specialized equipment, not widely available.
	Preoperative counseling	**Pro**: Personalized, reduces anxiety through education and reassurance. **Con**: Requires time, may not be feasible for all patients.
**Information-based**	Video-based Information Transfer	**Pro**: Reduces anxiety, saves time in preoperative consultations. **Con**: Requires technology access, may not address all patient concerns.
	Digital Platforms (e.g. VR, tutorials)	**Pro**: Tailored education, improves patient understanding and anxiety. **Con**: Needs resources for implementation, not always personalized enough.
**Special settings**	Pediatric Anesthesia	**Pro**: Special scales and child-friendly interventions can help reduce anxiety. **Con**: Limited pharmacologic options, validated child-specific tools.
	Day-Case Surgery	**Pro**: Shorter stay in hospital may be less stressful. **Con**: Anxiety underestimated in this setting.
